# Radiomic Analysis of Intrahepatic Cholangiocarcinoma: Non-Invasive Prediction of Pathology Data: A Multicenter Study to Develop a Clinical–Radiomic Model

**DOI:** 10.3390/cancers15174204

**Published:** 2023-08-22

**Authors:** Francesco Fiz, Noemi Rossi, Serena Langella, Andrea Ruzzenente, Matteo Serenari, Francesco Ardito, Alessandro Cucchetti, Teresa Gallo, Giulia Zamboni, Cristina Mosconi, Luca Boldrini, Mariateresa Mirarchi, Stefano Cirillo, Mario De Bellis, Ilaria Pecorella, Nadia Russolillo, Martina Borzi, Giulio Vara, Caterina Mele, Giorgio Ercolani, Felice Giuliante, Matteo Ravaioli, Alfredo Guglielmi, Alessandro Ferrero, Martina Sollini, Arturo Chiti, Guido Torzilli, Francesca Ieva, Luca Viganò

**Affiliations:** 1 Department of Nuclear Medicine, IRCCS Humanitas Research Hospital, 20089 Milan, Italy; francesco.fiz.nm@gmail.com (F.F.); martina.sollini@hunimed.eu (M.S.); arturo.chiti@hsr.it (A.C.); 2 MOX Laboratory, Department of Mathematics, Politecnico di Milano, 20133 Milan, Italy; noemi.rossi@mail.polimi.it (N.R.); francesca.ieva@polimi.it (F.I.); 3 Department of Digestive and Hepatobiliary Surgery, Mauriziano Umberto I Hospital, 10128 Turin, Italy; slangella@mauriziano.it (S.L.); nrussolillo@mauriziano.it (N.R.); aferrero@mauriziano.it (A.F.); 4 Division of General and Hepatobiliary Surgery, Department of Surgical Sciences, Dentistry, Gynecology and Pediatrics, University of Verona, University Hospital G.B. Rossi, 37134 Verona, Italy; ruzzenentea@gmail.com (A.R.); mario.debellis_01@univr.it (M.D.B.); alfredo.guglielmi@univr.it (A.G.); 5 General Surgery and Transplant Unit, IRCCS, Azienda Ospedaliero-Universitaria di Bologna, Sant’Orsola-Malpighi Hospital, 40138 Bologna, Italy; matteo.serenari@gmail.com (M.S.); matteo.ravaioli6@unibo.it (M.R.); 6 Department of Medical and Surgical Sciences, Alma Mater Studiorum, University of Bologna, 40126 Bologna, Italy; alessandro.cucchett2@unibo.it (A.C.); giorgio.ercolani2@unibo.it (G.E.); 7 Hepatobiliary Surgery Unit, A. Gemelli Hospital, Università Cattolica del Sacro Cuore, 00168 Rome, Italy; francesco.ardito@unicatt.it (F.A.); caterina.mele@policlinicogemelli.it (C.M.); felice.giuliante@unicatt.it (F.G.); 8 Department of General Surgery, Morgagni-Pierantoni Hospital, 47121 Forlì, Italy; mteresamirarchi@gmail.com; 9 Department of Radiology, Mauriziano Umberto I Hospital, 10128 Turin, Italy; tgallo@mauriziano.it (T.G.); scirillo@mauriziano.it (S.C.); 10 Department of Radiology, University of Verona, University Hospital G.B. Rossi, 37134 Verona, Italy; giulia.zamboni@aovr.veneto.it (G.Z.); martiborzi@gmail.com (M.B.); 11 Department of Radiology, IRCCS, Azienda Ospedaliero-Universitaria di Bologna, Sant’Orsola-Malpighi Hospital, 40138 Bologna, Italy; cristina.mosconi@aosp.bo.it (C.M.); giulio.vara@gmail.com (G.V.); 12 Department of Radiology, Radiation Oncology and Hematology, Fondazione Policlinico Universitario “A. Gemelli” IRCCS, 00168 Rome, Italy; luca.boldrini@policlinicogemelli.it; 13 Department of Biomedical Sciences, Humanitas University, Pieve Emanuele, 20072 Milan, Italy; ilariapecorella98@gmail.com (I.P.); guido.torzilli@hunimed.eu (G.T.); 14 Division of Hepatobiliary and General Surgery, Department of Surgery, IRCCS Humanitas Research Hospital, 20089 Rozzano, Italy; 15 CHDS—Center for Health Data Science, Human Technopole, 20157 Milan, Italy; 16 Hepatobiliary Unit, Department of Minimally Invasive General & Oncologic Surgery, Humanitas Gavazzeni University Hospital, 24125 Bergamo, Italy

**Keywords:** mass-forming intra-hepatic cholangiocarcinoma, microvascular invasion, grading, pathology data, prognosis, CT-based radiomics, advanced imaging and analyses, prediction of outcome, peritumoral tissue, biomarkers

## Abstract

**Simple Summary:**

Intrahepatic cholangiocarcinoma is a disease with increasing incidence and poor prognosis. The clinicians have a limited capability to predict tumor behavior because the strongest predictors of survival are the pathology data that, unfortunately, can be determined only after surgery. Recently, radiomics, i.e., the mathematical analysis of imaging modalities, led to a major improvement in the non-invasive prediction of microscopic characteristics of several tumors. In this multicenter study, we collected a large number of patients affected by intrahepatic cholangiocarcinoma and we demonstrated that the radiomic data of the tumor and peritumoral tissue extracted from the computed tomography at diagnosis have a strong association with tumor grading and microscopic vascular invasion, which are two major biomarkers of tumor aggressiveness. The combination of radiomic and clinical data maximizes the accuracy of prediction. The integration of radiomics into clinical decision processes is probably one of the following steps toward a precision medicine approach in patients affected by intrahepatic cholangiocarcinoma.

**Abstract:**

Standard imaging cannot assess the pathology details of intrahepatic cholangiocarcinoma (ICC). We investigated whether CT-based radiomics may improve the prediction of tumor characteristics. All consecutive patients undergoing liver resection for ICC (2009-2019) in six high-volume centers were evaluated for inclusion. On the preoperative CT, we segmented the ICC (Tumor-VOI, i.e., volume-of-interest) and a 5-mm parenchyma rim around the tumor (Margin-VOI). We considered two types of pathology data: tumor grading (G) and microvascular invasion (MVI). The predictive models were internally validated. Overall, 244 patients were analyzed: 82 (34%) had G3 tumors and 139 (57%) had MVI. For G3 prediction, the clinical model had an AUC = 0.69 and an Accuracy = 0.68 at internal cross-validation. The addition of radiomic features extracted from the portal phase of CT improved the model performance (Clinical data+Tumor-VOI: AUC = 0.73/Accuracy = 0.72; +Tumor-/Margin-VOI: AUC = 0.77/Accuracy = 0.77). Also for MVI prediction, the addition of portal phase radiomics improved the model performance (Clinical data: AUC = 0.75/Accuracy = 0.70; +Tumor-VOI: AUC = 0.82/Accuracy = 0.73; +Tumor-/Margin-VOI: AUC = 0.82/Accuracy = 0.75). The permutation tests confirmed that a combined clinical–radiomic model outperforms a purely clinical one (*p* < 0.05). The addition of the textural features extracted from the arterial phase had no impact. In conclusion, the radiomic features of the tumor and peritumoral tissue extracted from the portal phase of preoperative CT improve the prediction of ICC grading and MVI.

## 1. Introduction

Intrahepatic cholangiocarcinoma (ICC) is no longer a rare entity; to date, it is the second most common primary hepatic malignancy with rapidly increasing incidence [1,2]. It is an aggressive tumor resistant to standard cytotoxic chemotherapy regimens [3]. The only potentially curative approach is surgery [4]. However, recurrence frequently occurs and affects long-term survival, which ranges between 25% and 40% at five years after resection [4,5,6]. Even if standard morphological parameters (tumor size, number of foci, and suspicious nodal metastases) have a good association with postoperative survival, the pathology data (tumor grading, microscopic vascular invasion, and N status) are the strongest prognosticators [7,8,9]. Unfortunately, these cannot be determined before surgery. A reliable non-invasive preoperative assessment of pathology data would represent an enormous asset for the multidisciplinary team since it would allow the selection of candidates for resection and the indications to preoperative therapy in tumors with aggressive biology to be refined.

In the last decade, the extraction of quantitative information from imaging has kindled considerable interest. In particular, radiomics, defined as the high-throughput extraction of textural features from medical images, has gained momentum [10]. There is growing evidence that the “microscopic” pattern of both morphological and molecular imaging can identify the intrinsic characteristics of the target tissue, such as vascularity and cellular density [11,12]. For ICC, some preliminary studies have highlighted an association between the radiomic features extracted from computed tomography (CT) and magnetic resonance imaging (MRI) and the pathology characteristics [13]. However, the results for tumor grading and microscopic vascular invasion (MVI) are discordant. Further, all papers considered radiomic scores or signatures, i.e., a mathematical combination of textural features, that achieve the highest correlation with the outcome parameter but may discourage validation and reduce the applicability of data. Finally, the authors did not consider the peritumoral parenchyma, i.e., the liver/tumor interface where tumor progression occurs. Our group recently highlighted that including the radiomic features of the peritumoral region extracted from [18F]-fluorodeoxyglucose PET can improve the prediction of tumor grading and MVI of ICC [14].

This large multicenter study aims (1) to elucidate if the radiomics of ICC and its peritumoral tissue extracted from preoperative CT can contribute to the non-invasive prediction of tumor grading and MVI; (2) to analyze the predictive performance of a combined clinical–radiomic model in comparison with that of a pure clinical one (model development).

## 2. Materials and Methods

The institutional databases of six high-volume centers were retrospectively analyzed to identify all consecutive patients affected by mass-forming ICC undergoing liver resection between January 2009 and December 2019. The complete list of the recruiting centers is reported in Supplementary Table S1. The following inclusion criteria were applied: adult subjects (≥18 years); diagnosis of ICC confirmed at final pathology; availability of a preoperative contrast-enhanced CT with an adequate portal venous phase (acquired within 70 to 90 s after contrast administration); and an interval between imaging and surgery ≤ 60 days. We included exclusively multislice CT with a maximum slice thickness of 5 mm. We utilized the following exclusion criteria: mixed ICC/hepatocellular carcinoma; tumor size < 10 mm (such a small size could be inadequate for some radiomic indices and peritumoral tissue analysis); low-quality imaging; surgery without liver resection (i.e., explorative laparotomy); and previous loco-regional treatments, including ablation or trans-arterial embolization. Neoadjuvant chemotherapy was not an exclusion criterion but the CT after treatment was considered. If no CT after the end of treatment and before surgery was performed, the patient was excluded. In the case of preoperative portal vein embolization, the pre-embolization images were analyzed.

The study was performed according to the declaration of Helsinki and its subsequent amendments. The protocol was reviewed and approved by the ethics committee of each participating center (for the coordinating center: protocol number 142/21, date of approval 17 March 2021). Because of the retrospective nature of the investigation, the need to obtain specific consent was waived. The study followed the TRIPOD guidelines (Supplementary Table S2).

### 2.1. Study Endpoints

The main objective of the study was (1) to build a predictive model of the tumor grading (G3 vs. G1-2) and MVI (present vs. absent) combining the clinical data with the radiomic indices of the tumor and peritumoral tissue extracted from the portal phase of the CT; (2) to compare the predictive performance of the combined clinical–radiomic model with that of a purely clinical model. The secondary endpoints were (1) to analyze the contribution of the radiomic data extracted from the arterial phase of CT; (2) to evaluate if the center of origin influences the association between the radiomic features and outcomes (tumor grading and MVI). 

### 2.2. Imaging Acquisition, Tumor Segmentation, and Radiomic Features Extraction

Even if the images were acquired on different devices, all centers applied a similar standardized protocol to perform CT for ICC staging, i.e., multiphasic breath-hold acquisitions including a pre-contrast phase, an automatically bolus-triggered arterial phase, a portal phase (acquired within 70 to 90 s after contrast administration), and an equilibrium (late) phase (about 3 min after contrast administration). 

The segmentation was performed in every participating center by a radiologist with a long-standing experience in image analysis using the LifeX software application. The following precautions were adopted to ensure a consistent segmentation procedure across all centers: (1) every institution utilized the same version of the software (version 6.3); (2) a technical meeting was held before the analysis, during which a consensus was reached about the procedure; and (3) the segmentation of the first two enrolled patients in each collaborating center was performed under the scrutiny of a physician from the coordinating institution.

**Figure 1:**
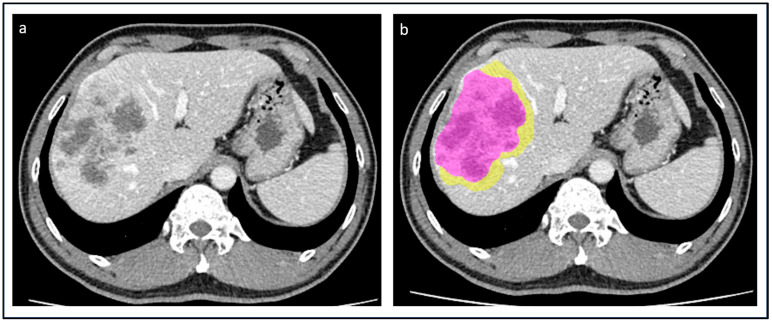
Patient with a mass-forming ICC of the right liver. (**a**) The tumor was identified on the portal phase of the CT; (**b**) manual segmentation of the tumor (violet area) and automatic segmentation of a 5 mm rim of peritumoral tissue (yellow area) were performed.

In subjects bearing multifocal ICC, only the largest lesion was analyzed. The tumor volume segmentation (Tumor-VOI) was performed manually on the portal phase (Figure 1). After that, the tumor volume was automatically expanded by 5 mm to generate a second VOI, representing the peritumoral tissue (Margin-VOI). A width of 5 mm was defined a priori to include a sufficient sample of tissue adequate for every tumor size. Every VOI was visually checked by physicians and manually corrected if needed. Both VOIs were then transferred to the arterial phase; if needed, the placement was manually corrected. 

The radiomic features were extracted using the LifeX program [15] and were adherent to the IBSI standard [16]. The full output of the analysis included 95 indices. Redundant values, nuclear medicine parameters, cardiology-specific scores, “rim” values, discretized indices, and technical information parameters were excluded. Forty-five indices were retained for the analysis, including seven standard grey-level descriptors (HUmin, HUmean, HUstd, HUmax, and the HU tertiles), four first-order parameters (skewness, kurtosis, histogram_energy, and histogram_entropy), three shape-related indices (volume, sphericity, and compacity), seven grey-level co-occurrence matrices (GLCM), eleven grey-level run-length matrices (GLRLM), three neighboring grey-level difference matrices (NGLDM), and eleven grey-level zone-length matrices (GLZLM). 

### 2.3. Statistical Analyses

After merging the databases, outliers and missing data were analyzed. All outliers were verified by sending queries to the participating centers and corrected if needed. The missing values were managed as follows: all patients with missing values of the radiomic features or the study endpoints (tumor grading or MVI) were excluded; for the remaining variables, multiple imputation analysis was performed by Python miceforest software with the ImputationKernel function if the proportion of missing data was <15% of cases. 

Categorical variables were compared with the chi-square or Fisher’s exact tests as appropriate. Continuous variables were assessed graphically to determine distribution normality and then compared with parametric (unpaired T-test) or non-parametric (Mann–Whitney U-test) tests. Continuous variables were included as predictors to preserve and maximize their predictive contribution. 

A multivariate logistic regression model was performed to estimate the adjusted association between each candidate predictor and (1) the tumor grading (G3 vs. G1-2), and (2) the MVI (present vs. absent). The clinical variables were selected according to a priori knowledge [6,7,8,9] and the results of univariate analysis (*p*-value < 0.1). The following variables were considered: demographic data (age and gender), HBV/HCV infection, liver cirrhosis, tumor characteristics (number, maximum diameter, and tumor pattern according to Baheti et al. [17]), preoperative CA 19-9 value, administration of preoperative chemotherapy, and type of scheduled resection (major/minor hepatectomy). The tumor pattern combines the number and distribution of tumors as follows: pattern type 1, solitary tumor; pattern type 2, multiple tumors into a single segment (including satellite nodules); pattern type 3, multiple tumors into different segments. 

All radiomic features were initially considered. For their selection and inclusion into the model, the correlation between features was evaluated, and if >0.85, one of the two features was removed. The radiomic indices of the Tumor-VOI and Margin-VOI were assessed independently. 

Different multivariate logistic regression models were tested (i.e., lasso logistic regression, ridge logistic regression, logistic regression with stepwise feature selection, logistic regression with forward feature selection, and logistic regression with backward feature selection). After the identification of the best model without accounting for the enrolment center (logistic regression with backward selection), the center effect was analyzed, fitting a suitable Mixed Effect Model (MEM) [18]. The center effect was evaluated by computing the Variance Partition Coefficient (VPC) [19]. 

The final predictive model underwent internal stratified K-fold cross-validation with k = 50. The results were reported in terms of mean (Std Dev) accuracy. Other machine learning methods were tested (e.g., CART, Random Forest), but the ratio between the sample size and the number of available features did not guarantee the stability of the results. Permutation tests (one-sided) were used to compare the average performance of the different models. 

A *p*-value < 0.05 was considered significant for all the tests. The analyses were carried out using STATA for Windows (StataCorp. 2019. Stata Statistical Software: Release 16. College Station, TX, USA: StataCorp LLC), R (R Core Team 2021), and Python (Version 3).

## 3. Results

Two hundred and sixty-six patients were recruited across the six participating centers (median 37, IQR 29-74). According to the inclusion criteria, 244 patients were retained (51% females, median age 67.5 years). Of those, 215 (88%) had an adequate arterial phase of CT. Patients’ characteristics are summarized in Table 1. In total, 82 (34%) patients had a G3 tumor, and 139 (57%) had ICC with MVI. The univariate association of clinical variables with tumor grading and MVI are detailed in Supplementary Table S3.

### 3.1. Prediction of Tumor Grading (G3 vs. G1-2)

**Figure 2:**
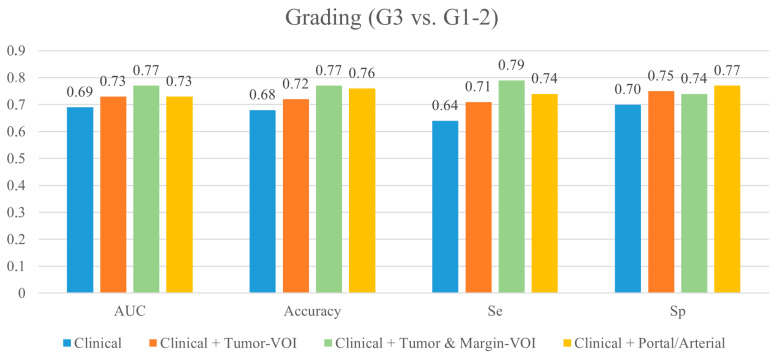
Performance of the best predictive models for tumor grading at the internal cross-validation. Se: sensitivity; Sp: specificity.

The models and their performances are detailed in Table 2, Figure 2, and Supplementary Table S4. At cross-validation, after correcting for the center’s effect, the model based on clinical data had the following performances: AUC = 0.694 ± 0.310, Sensitivity = 0.635 ± 0.422, Specificity = 0.698 ± 0.210, and Accuracy = 0.680 ± 0.239. No variable had a significant association with the outcome.

After including the Tumor-VOI radiomics extracted from the portal phase of CT, the model’s AUC, sensitivity, specificity, and accuracy increased to 0.731 ± 0.276, 0.706 ± 0.384, 0.745 ± 0.203, and 0.720 ± 0.222, respectively. One radiomic variable—GLRLM_SRHGE—was retained in the model (OR = 0.733). When the radiomic features extracted from the Margin-VOI were added (clinical variables + Tumor-VOI and Margin-VOI radiomics), the AUC, sensitivity, and accuracy further increased (0.767 ± 0.270, 0.789 ± 0.339, and 0.765 ± 0.182, respectively), while specificity did not (0.741 ± 0.195). The final model included one clinical variable (major hepatectomy, OR = 1.661), two Tumor-VOI features (HUmin, OR = 1.521; and GLRLM_SRHGE, OR = 0.672), and two Margin-VOI features (NGLDM_Busyness, OR = 0. 644; and GLZLM_ZLNU, OR = 2.050).

The permutation test, comparing the mean performances of the tested models, demonstrated that the addition of the Tumor-VOI radiomic features to the clinical data improved the whole performance of the clinical model (*p* < 0.05, Table 3) and that the further addition of the Margin-VOI radiomic features led to a further global improvement (*p* < 0.05).

The addition of the radiomic features extracted from the arterial phase (Tumor-VOI and Margin-VOI, 215 patients) did not grant an improvement in the model performance (AUC = 0.730 ± 0.302, Sensitivity = 0.740 ± 0.327, and Accuracy = 0.760 ± 0.194) except for a slight increase in the specificity (0.766 ± 0.215) (Supplementary Table S4). At the permutation test, only the increase in specificity was significant (*p* < 0.05).

The VPC values were as follows: 25.9% for the pure clinical model, 30.0% for the model combining clinical data and Tumor-VOI radiomics extracted from the portal phase, 26.6% for the model combining clinical data and Tumor- and Margin-VOI radiomics derived from the portal phase, and 13.9% for the model including the radiomic features extracted from the arterial phase.

### 3.2. Prediction of Microscopic Vascular Invasion

**Figure 3:**
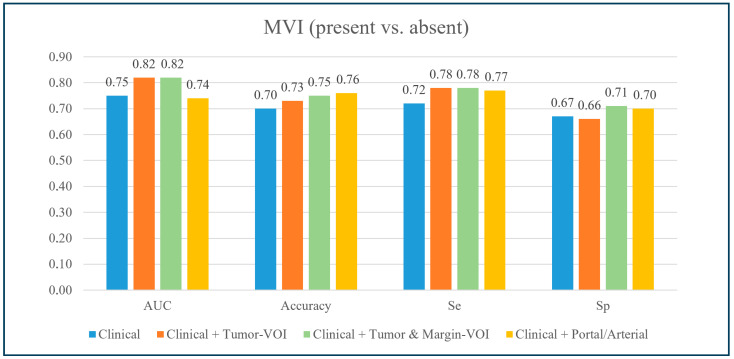
Performance of the best predictive models for microscopic vascular invasion at the internal cross-validation. Se: sensitivity; Sp: specificity.

The models and their performances are detailed in Table 4, Figure 3, and Supplementary Table S5. At cross-validation, after correcting for the center’s effect, the model based on clinical data (age, Ca 19-9 value, and major hepatectomy) had the following performances: AUC = 0.752 ± 0.298, Sensitivity = 0.717 ± 0.302, Specificity = 0.673 ± 0.354, and Accuracy = 0.696 ± 0.249.

After including the Tumor-VOI radiomics extracted from the portal phase of CT, the model’s AUC, sensitivity, and accuracy increased (0.821 ± 0.236, 0.775 ± 0.294, and 0.725 ± 0.216, respectively), while specificity did not (0.655 ± 0.346). The model retained three variables: major hepatectomy (OR = 3.277), HUmin (OR = 0.496), and GLRLM_SRHGE (OR = 0.673). When adding the radiomic features of the Margin-VOI (clinical data + portal Tumor- and Margin-VOI radiomics), the model’s AUC and sensitivity did not show a further improvement (0.823 ± 0.230 and 0.777 ± 0.271, respectively), while specificity increased to 0.714 ± 0.340. Overall accuracy reached 0.753 ± 0.236. The model retained one clinical variable (major hepatectomy, OR = 2.760) and five Margin-VOI features (HUQ2, OR = 0.651; Shape_Sphericity, OR = 0.560; GLCM_Correlation, OR = 1.542; NGLDM_Contrast, OR = 1.436; and GLZLM_SZHGE, OR = 1.636).

At permutation test, comparing the mean performances of the tested models, the Tumor-VOI radiomics extracted from the portal phase of the CT led to an AUC improvement, while the addition of both Tumor-VOI and Margin-VOI radiomics improved all performances in comparison with the clinical model and accuracy, specificity, and sensitivity in comparison with the clinical + Tumor-VOI model (*p* < 0.05, Table 5).

The addition of the radiomic features extracted from the arterial phase of the CT (Supplementary Table S5) did not modify the model performances (AUC: 0.744 ± 0.285, Sensitivity: 0.765 ± 0.301, and Specificity: 0.703 ± 0.285), except for a slight improvement in the accuracy (0.759 ± 0.201). The permutation test confirmed the increase in accuracy as the sole improvement achieved by adding the arterial phase radiomics (*p* < 0.05).

The VPC values were as follows: 21.2% for the pure clinical model, 24.4% for the model combining clinical data and Tumor-VOI radiomics extracted from the portal phase, 19.8% for the model combining clinical data and Tumor- and Margin-VOI radiomics extracted from the portal phase, and 14.5% for the model including also the radiomic features extracted from the arterial phase.

## 4. Discussion

The present study demonstrates that the radiomic analysis of the tumor and peritumoral tissue may predict the ICC grading and MVI. The features extracted from the portal phase play a crucial role, while the ones from the arterial phase do not. The combined clinical–radiomic model achieves the best predictive performance, even if a center’s effect should be considered.

Surgery is the standard treatment of ICC, but some patients with aggressive tumors do not benefit from resection and experience early recurrence and early cancer-related death [6,8,9,20]. Tsilimigras et al. published a score based on the tumor size and number and the clinical suspicion of lymph node metastases to predict early surgical failure [20]. High-risk patients should probably be considered for systemic therapy before any loco-regional treatments. However, morphological parameters are a poor surrogate of tumor biology and do not guarantee adequate selection of patients. Conci et al. demonstrated that the ICC distribution rather than their number is crucial for prognosis and that surgery remains the mainstay approach in patients with multifocal but localized disease [6]. The pathology data are the ground truth of tumor biology and should drive treatment planning. Among them, tumor grading and MVI are strong predictors of outcome [7,8,9]. The vascular invasion is a key parameter in the TNM staging system, even more relevant than the number of tumor foci [21].

The capability of radiomics to predict pathology data has been the object of several publications for many tumors, including ICC [11,13,22]. The strongest evidence concerns lymph node metastases [23,24,25,26]. Data about MVI and tumor grading are less robust. The radiomic analysis based on preoperative CT [27] and MRI [28,29] guarantees an adequate prediction of the MVI status (AUC range 0.76–0.85), but its clinical impact is unclear. While Qian et al. reported that a combined radiological–radiomic model outperforms a pure clinical one (AUC 0.95 vs. 0.73) [29], Xiang et al. did not confirm such results [27]. The data about tumor grading are even less favorable: Peng et al. observed poor performances of radiomics (AUC = 0.71) [30], and King et al. observed no association [31]. Additional limitations of the available papers deserve consideration: all studies have been published by Eastern centers and adopted signatures or scores combining multiple features to maximize the performance of radiomics. The present series collected a large number of patients (n = 244) with a high proportion of G3 and MVI+ tumors (one-third and half of patients, respectively) and kept the single radiomics features apart, leading to a complete and easy replicability of data.

In the present study, the pure clinical models showed unsatisfactory performances for tumor grading (AUC < 0.70 without any significant predictors) and yielded intermediate results for MVI (AUC = 0.75). The addition of the radiomic features led to a substantial improvement in the predictive performances for both outcomes (tumor grading, AUC = 0.73/Accuracy = 0.72; MVI, AUC = 0.82/Accuracy = 0.73), and the results were maximized when both the tumor and margin were considered (AUC = 0.77/Accuracy = 0.77 and AUC = 0.82/Accuracy = 0.75, respectively). The permutation test, which compares the mean performance of the predictive models, confirmed that the clinical–radiomic models outperform the clinical ones and that the combined analysis of the tumor and its margin outperforms the analysis of the tumor. The peritumoral tissue is the niche of relevant biomarkers, such as micrometastases, satellite nodules, and immune infiltrate [32,33,34]. Yugawa et al. reported some associations between the radiomics of the peritumoral tissue and immune infiltrate [35]. In the present series, only margin-related features were retained in the final predictive models of MVI, reflecting that vascular invasion plays a key role in tumor progression at the liver–tumor interface.

Looking at the final models, we may suggest some correlations between radiomic and clinical data. High tumor grading (G3) was associated with portal contrast enhancement (higher HUmin values), as well as with the inhomogeneous texture of both the tumor (lower GLRLM_SRHGE) and peritumoral tissue (lower NGLDM_Busyness and higher GLZLM_ZLNU). This pattern may reflect the presence of active tissue (as confirmed at PET-CT evaluation [14]), peripheral neoangiogenesis, and necrotic areas. For MVI, the final model retained four margin-related radiomic features: an irregular shape (lower Sphericity) and coarseness of texture (higher GLCM_Correlation, GLZLM_SZHGE, and NGLDM_Contrast) were associated with vascular invasion. Analogously, a previous study by Fiz et al. demonstrated that higher values of NGLDM and GLRLM indices in the peritumoral tissue at PET-CT predict MVI [14]. Even if further investigations are still required in this clinical setting, it appears plausible that, as observed for colorectal liver metastases, an infiltrative tumor profile and a more irregular peritumoral tissue could be associated with higher tumor aggressiveness [36,37].

We also explored the contribution of the radiomic features extracted from the arterial phase of the CT. ICC has specific patterns of enhancement in the arterial phase, which have been associated with tumor biology and prognosis [38,39,40,41]. In the present analysis, the radiomic features extracted from the arterial phase made a marginal contribution to the models. Xiang et al. reported similar results: despite the radiomic analysis of both the arterial and portal phase, the latter provided the best predictors of MVI [27]. Even if further studies are needed to address such discrepancy, we may advance some hypotheses. Firstly, the radiomics of the portal phase could provide the full information. Secondly, in a retrospective setting, the arterial phase may suffer from a higher heterogeneity than the portal one.

The present study is clinically relevant. The planning of treatment strategies for aggressive diseases should rely on the accurate evaluation of tumor biology. Radiomics allows a non-invasive prediction of the tumor grading and MVI, two highly relevant biomarkers, and anticipates in the preoperative setting information usually available only at the pathology analysis after surgery. In patients with borderline resectable ICC, the presence of radiomic markers linked with an aggressive disease could be used as a possible criterion to indicate a neoadjuvant treatment instead of upfront surgery. We obtained such favorable results in a multicenter setting with unselected CT in a real-life scenario.

The present results are in line with the ones we previously obtained in a monocentric analysis considering ICC and PET-based radiomics. Some radiomic indices were even identified as predictors of pathology data in both studies. Because of the different study design, we cannot compare the radiomics extracted from the two imaging modalities (CT and PET) in terms of clinical benefit and usefulness. To date, CT and PET/CT should be performed according to their clinical indications and, in the authors’ opinion, both are needed in ICC patients who are candidates for surgery. In the near future, the availability of fusion imaging (PET/CT contrast enhanced) and AI-based protocols applied to medical imaging will lead to an easy extraction and integration of radiomics from multiple imaging modalities, providing a unique and complete imaging-based profiling of ICC.

The transferability of present data into clinical practice needs some further steps. Firstly, external validation is necessary; such validation should be prospective, multicentric, and performed across international HPB units to definitively take radiomics to the bedside [42]. Secondly, the impact of the center’s effect needs some further investigation. The data originated from different institutions and thus from different CT devices. The standardization of CT protocols remains an issue, but major efforts are ongoing to harmonize data from different image acquisitions [16]. Thirdly, segmentation was performed manually, which is a time-consuming procedure. Available semi-automatic procedures still require major manual corrections; the inclusion of the peritumoral rim further adds complexity. In the near future, AI-powered automatic segmentation protocols will speed up the process and ease the inclusion of radiomics into clinical practice. Finally, clear cut-off values of the radiomic features should be identified to include the variables in the clinical models.

## 5. Conclusions

CT-based radiomic features extracted from the tumor and its margin provide an accurate non-invasive prediction of tumor aggressiveness that standard clinical parameters cannot reach. Integrating radiomics into clinical decision processes is probably one of the next steps toward a precision medicine approach in ICC patients.

## Figures and Tables

**Table 1:** Patient’s characteristics. Tumor pattern was defined according to Baheti et al. [17] as follows: pattern type 1, solitary tumor; pattern type 2, multiple tumors into a single segment (including satellite nodules); pattern type 3, multiple tumors into different segments.

Characteristic	Number (%)—Median (Range)	Missing (Number)
Age, years	67.5 (21–86)	-
Sex, male/female	120 (49.2%):124 (50.8%)	-
HBV infection	19 (7.8%)	1
HCV infection	27 (11.1%)	1
Liver cirrhosis	26 (10.7%)	-
Tumor diameter, mm	50 (10–270)	-
Solitary tumor	206 (84.4%)	-
Tumor pattern		
Type 1	151 (61.9%)	-
Type 2	61 (25.0%)	-
Type 3	32 (13.1%)	-
Ca 19.9, U/mL	29 (0.2–67,456.3)	30
Ca 19.9 ≥ 55 U/mL	74 (30.3%)	30
Preoperative chemotherapy	26 (10.7%)	-
Partial response	13 (50%)	3
Stable disease	8 (30.8%)	
Disease progression	2 (7.7%)	
Major hepatectomy	128 (52.5%)	-
Tumor grading, G3	82 (33.6%)	-
Microscopic vascular invasion	139 (57%)	-

**Table 2:** Multivariate analyses of preoperative predictors of tumor grading (G3 vs. G1-2). Tumor pattern was defined according to Baheti et al. [17] as follows: pattern type 1, solitary tumor; pattern type 2, multiple tumors into a single segment (including satellite nodules); pattern type 3, multiple tumors into different segments.

Parameter	Odds Ratio	Lower Bound	Upper Bound	*p*-Value
**Model with preoperative clinical data**				
Age (years)	1.080	0.786	1.480	0.638
Sex	1.550	0.837	2.850	0.164
HBV	0.614	0.177	2.120	0.441
HCV	1.690	0.647	4.440	0.283
CA 19-9 (ng/mL)	1.110	0.803	1.530	0.528
Preoperative chemotherapy	1.310	0.505	3.380	0.582
Major hepatectomy	1.490	0.754	2.940	0.251
Cirrhosis	0.947	0.348	2.580	0.916
Tumor pattern				
Type 1	1	-	-	-
Type 2	1.200	0.541	2.640	0.658
Type 3	1.800	0.354	9.160	0.478
Tumor size (mm)	1.230	0.889	1.690	0.214
Single nodule	1.440	0.318	6.470	0.638
**Model with preoperative clinical data + Tumor-VOI radiomics (portal phase)**				
Portal_Tumor_GLRLM_SRHGE	0.733	0.556	0.966	0.027
**Model with preoperative clinical data + Tumor- and Margin-VOI radiomics (portal phase)**				
Major hepatectomy	1.661	1.132	2.438	0.010
Portal_Tumor_HUmin	1.521	0.944	2.453	0.085
Portal_Tumor_GLRLM_SRHGE	0.672	0.497	0.908	0.010
Portal_Margin_NGLDM_Busyness	0.644	0.442	0.938	0.022
Portal_Margin_GLZLM_ZLNU	2.050	1.284	3.274	0.003

**Table 3:** Results of the permutation tests for tumor grading.

	Clinical vs. Tumor-VOI	Clinical vs. Tumor- and Margin-VOI	Tumor-VOI vs. Tumor- and Margin-VOI
Tumor Grading			
Accuracy	0.007	<0.001	<0.001
Specificity	0.035	<0.001	0.037
Sensitivity	0.028	<0.001	<0.001
Precision	0.003	<0.001	0.118
Pr AUC	<0.001	<0.001	<0.001
Roc AUC	0.476	<0.001	<0.001

**Table 4:** Multivariate analyses of preoperative predictors of MVI.

Parameter	Odds Ratio	Lower Bound	Upper Bound	*p*-Value
**Model with preoperative clinical data**				
Age (years)	0.992	0.987	0.998	0.007
CA 19-9 (ng/mL)	1.001	1.000	1.001	0.056
Major hepatectomy	3.296	1.934	5.617	<0.001
**Model with preoperative clinical data + Tumor-VOI radiomics (portal phase)**				
Major hepatectomy	3.277	2.180	4.925	<0.001
Portal_Tumor_HUmin	0.496	0.307	0.800	0.004
Portal_Tumor_GLRLM_SRHGE	0.673	0502	0.902	0.008
**Model with preoperative clinical data + Tumor- and Margin-VOI radiomics (portal phase)**				
Major hepatectomy	2.760	1.820	4.186	<0.001
Portal_Margin_HUQ2	0.651	0.460	0.922	0.015
Portal_Margin_Shape_Sphericity	0.560	0.408	0.768	<0.001
Portal_Margin_GLCM_Correlation	1.542	1.112	2.139	0.009
Portal_Margin_NGLDM_Contrast	1.436	0.924	2.231	0.107
Portal_Margin_GLZLM_SZHGE	1.636	1.043	2.567	0.032

**Table 5:** Results of the permutation tests for MVI.

	Clinical vs. Tumor-VOI	Clinical vs. Tumor- and Margin-VOI	Tumor-VOI vs. Tumor- and Margin-VOI
**Microscopic vascular invasion**			
Accuracy	0.295	<0.001	0.001
Specificity	0.072	<0.001	0.025
Sensitivity	0.152	<0.001	0.008
Precision	0.081	0.003	0.087
Pr AUC	0.014	0.008	0.212
Roc AUC	0.007	<0.001	0.112

## Data Availability

The datasets generated during and/or analyzed during the current study are available from the corresponding author on reasonable request.

## Supplementary Materials

The following supporting information can be downloaded at: https://www.mdpi.com/article/10.3390/cancers15174204/s1, Table S1: List of the participating centers; Table S2: The TRIPOD checklist; Table S3: Univariate analysis of clinical predictors of G3 ICC and MVI; Table S4: Multivariate analyses of preoperative predictors of tumor grading (G3 vs. G1-2). Model with preoperative clinical data + Tumor- and Margin-VOI radiomics (portal and arterial phases); Table S5: Multivariate analyses of preoperative predictors of MVI. Model with preoperative clinical data + Tumor- and Margin-VOI radiomics (portal and arterial phases).
